# Computational Investigation of the Changing Patterns of Subtype Specific NMDA Receptor Activation during Physiological Glutamatergic Neurotransmission

**DOI:** 10.1371/journal.pcbi.1002106

**Published:** 2011-06-30

**Authors:** Pallab Singh, Adam J. Hockenberry, Vineet R. Tiruvadi, David F. Meaney

**Affiliations:** Department of Bioengineering, University of Pennsylvania, Philadelphia, Pennsylvania, United States of America; University of Virginia, United States of America

## Abstract

NMDA receptors (NMDARs) are the major mediator of the postsynaptic response during synaptic neurotransmission. The diversity of roles for NMDARs in influencing synaptic plasticity and neuronal survival is often linked to selective activation of multiple NMDAR subtypes (NR1/NR2A-NMDARs, NR1/NR2B-NMDARs, and triheteromeric NR1/NR2A/NR2B-NMDARs). However, the lack of available pharmacological tools to block specific NMDAR populations leads to debates on the potential role for each NMDAR subtype in physiological signaling, including different models of synaptic plasticity. Here, we developed a computational model of glutamatergic signaling at a prototypical dendritic spine to examine the patterns of NMDAR subtype activation at temporal and spatial resolutions that are difficult to obtain experimentally. We demonstrate that NMDAR subtypes have different dynamic ranges of activation, with NR1/NR2A-NMDAR activation sensitive at univesicular glutamate release conditions, and NR2B containing NMDARs contributing at conditions of multivesicular release. We further show that NR1/NR2A-NMDAR signaling dominates in conditions simulating long-term depression (LTD), while the contribution of NR2B containing NMDAR significantly increases for stimulation frequencies that approximate long-term potentiation (LTP). Finally, we show that NR1/NR2A-NMDAR content significantly enhances response magnitude and fidelity at single synapses during chemical LTP and spike timed dependent plasticity induction, pointing out an important developmental switch in synaptic maturation. Together, our model suggests that NMDAR subtypes are differentially activated during different types of physiological glutamatergic signaling, enhancing the ability for individual spines to produce unique responses to these different inputs.

## Author Summary

Release of glutamate from one neuron onto glutamate receptors on adjacent neurons serves as the primary basis for neuronal communication. Further, different types of glutamate signals produce unique responses within the neuronal network, providing the ability for glutamate receptors to discriminate between alternative types of signaling. The NMDA receptor (NMDAR) is a glutamate receptor that mediates a variety of physiological functions, including the molecular basis for learning and memory. These receptors exist as a variety of subtypes, and this molecular heterogeneity is used to explain the diversity in signaling initiated by NMDARs. However, the lack of reliable experimental tools to control the activation of each subtype has led to debate over the subtype specific roles of the NMDAR. We have developed a stochastic model of glutamate receptor activation at a single synapse and find that NMDAR subtypes detect different types of glutamate signals. Moreover, the presence of multiple populations of NMDAR subtypes on a given neuron allows for differential patterns of NMDAR activation in response to varied glutamate inputs. This model demonstrates how NMDAR subtypes enable effective and reliable communication within neuronal networks and can be used as a tool to examine specific roles of NMDAR subtypes in neuronal function.

## Introduction

Synaptic neurotransmission in excitatory neural circuits is governed primarily by the activation of AMPA receptors (AMPARs) and NMDA receptors (NMDARs), two types of ionotropic glutamate receptors located on dendritic spines. Although AMPARs are critical in mediating action potential firing through neuronal networks, NMDARs are often more critical in adaptation of the network during neuronal development [1], [2], learning, and memory [3], [4], [5], [6]. Moreover, recent evidence shows activation of synaptic NMDA receptors is essential for proper health and maintenance of the neuronal network [7], [8], [9]. In contrast, persisting high levels of NMDAR activation leads to the induction of numerous signaling pathways that contribute to neuronal death and loss of network function [10], [11], [12], [13]. Therefore, activation of NMDARs is a precise balancing act that can control the function and integrity of *in vivo* and *in vitro* neural circuits.

Recent evidence points to the molecular composition of the NMDAR as a possible critical point for regulating the influence of NMDAR activation in networks. Functional NMDARs are expressed on the neuronal surface as a tetramer, comprised of 2 NR1 subunits and 2 subunits from the NR2 family (NR2A, NR2B, NR2C, and NR2D) [14], [15]. The NR2A and NR2B subunit expression dominates in the cortex and hippocampus, with past work showing functional NMDARs are expressed either in a diheteromeric (NR1/NR2A, NR1/NR2B) or triheteromeric form (NR1/NR2A/NR2B) [14], [16]. Moreover, the NMDAR composition changes through development, with one diheteromeric form (NR1/NR2B) dominating in immature neurons, eventually augmented by NR2A-containing NMDARs at synaptic sites [16],[17],[18]. The molecular composition of the receptor, as well as its location, can regulate synaptic plasticity [19], [20], [21], receptor trafficking [22], and the activation of specific synaptic signaling networks [23], [24]. More recent reports show that regulation of synaptic changes can be confined to one or a few individual spines, suggesting a need to understand the broad diversity in glutamate receptor signaling that occurs in individual spines [25], [26]. However, developing a more precise relationship between presynaptic glutamate release and the activation of specific NMDAR subtypes on individual synapses is difficult and technically demanding. Ongoing discussions in the literature and the considerable limitations and caveats of current pharmacological manipulations of individual subtypes [27] have created the need for alternative methods to better examine the activity of specific NMDAR subtypes.

Computational modeling offers an alternative approach for examining the relative balance of NMDAR activation in single spines, with past simulations of glutamatergic signaling used to investigate synaptic communication at temporal and spatial resolutions that are difficult or impossible to study experimentally. The stochastic nature of glutamate receptor activation [28], [29] and their contribution to the quantal properties of synaptic transmission [29], [30] reveal the conditions necessary for receptor saturation and explain variation in postsynaptic response. Further investigation into the role of glutamate uptake [31], [32] and spillover [32], [33], [34] identify their critical roles in modulating the activation profiles at neighboring synapses. The development of NMDAR subtype specific reaction schemes [35] extend the utility of computational models to investigate the differences in activation of different NMDAR subtypes, with a recent study demonstrating the greater probability of activation of NR1/NR2A-NMDARs compared to NR1/NR2B-NMDARs and the role of different subtypes in mediating downstream signaling [36]. Less well described, though, is how synaptic signaling through NMDARs may provide a mechanism to scale synaptic inputs over the physiological range, and how the relative composition of NMDARs on the postsynaptic surface may shape the scaling of the NMDAR response over conditions that span long-term depression (LTD) and long-term potentiation (LTP). Moreover, little is known about how neuronal development influences NMDAR signaling, and if these changes in neuronal development will shift the NMDAR-based signaling from one receptor subpopulation to another.

In this paper, we use computational simulations to examine how NMDAR subtype and overall NMDAR content of the dendritic spine can impact the extent and reliability of synaptic transmission. Further, we determine how the unique properties of activation among NMDAR subtypes create distinct activation patterns among synapses with differing compositions. We show that NR2A-containing NMDARs provide the most dynamic range across univesicular and, to a lesser extent, multivesicular release conditions. Alternatively, the NR2B-containing NMDARs play a larger role in simulated multivesicular release conditions, and contribute more significantly to the NMDAR input during high frequency stimulation. These data are supported by past studies in the literature, and illustrate how the existence of multiple NMDAR subpopulations at individual spines enables the efficient transduction of a wide variety of glutamate signals into unique postsynaptic responses.

## Results

**Figure 1:**
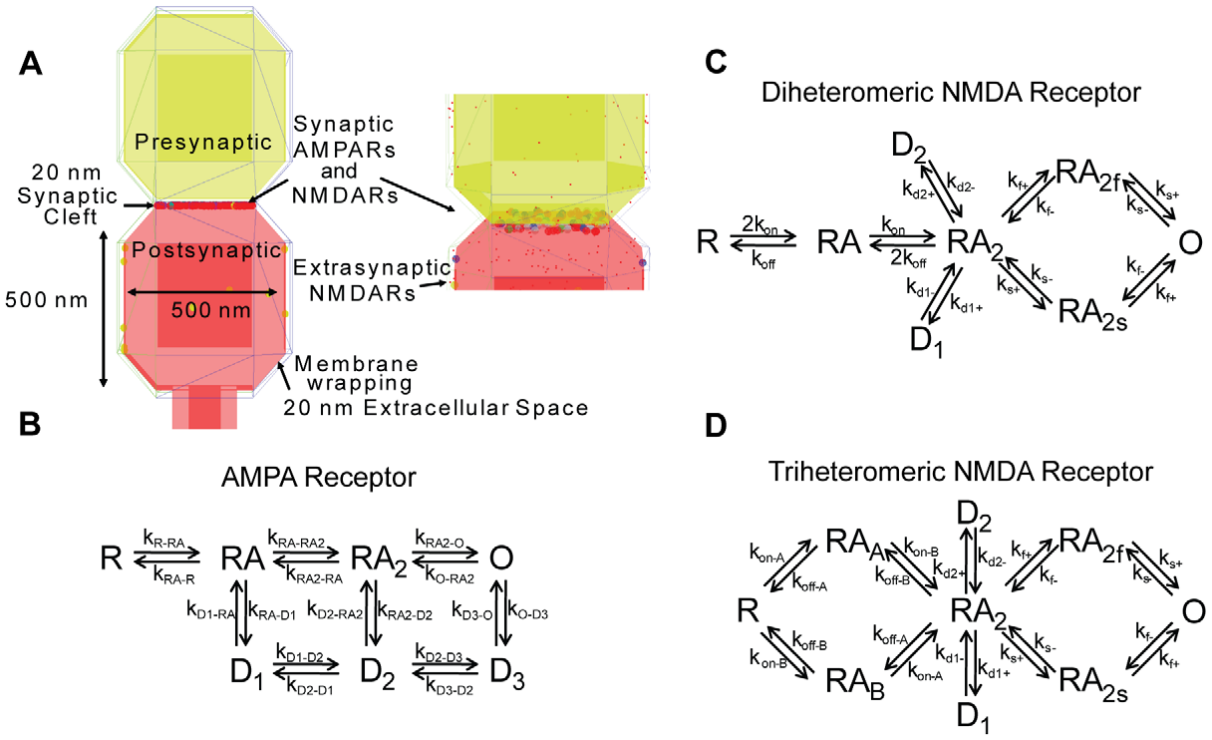
Dendritic spine model geometry and receptor activation schemes. (A) Representation of the computational model of the dendritic spine, which includes a 20 nm synaptic cleft. The postsynaptic compartment contains synaptic AMPARs and NMDARs and extrasynaptic NMDARs. Activation of glutamate receptors were determined using previously established reaction schemes for (B) AMPARs, (C) NR2A-NMDARs and NR2B-NMDARs, and (D) triheteromeric NR2A/NR2B-NMDARs. Constants used in the reaction schemes are provided in Table 1. Simulations tracked all receptor states, and reported the fraction of open receptors.

We created a stochastic model of glutamatergic signaling at the dendritic spine to study the differences in NMDAR subtype activation among several physiological conditions. We used Smoldyn (Version 1.84) [37], [38], a spatial stochastic simulator for biochemical reaction networks, and developed a model using the typical dimensions of a mature, thin spine [39] (Figure 1A). With the understanding that activity across multiple types of synapses throughout the brain can vary significantly, in these studies we intended to examine receptor activation at a prototypical synapse to broaden the applicability of our results. We utilized previously published reaction schemes (Figure 1B,C,D) for the activation of specific NMDAR subtypes [35] and AMPARs [40] (see methods for more details). We restricted nearly all of our analysis to the open state for each receptor, defined when glutamate is bound to receptor subunits and has transitioned into an activated state. We studied three primary aspects of synaptic signaling with this model: the scaling and relative activation of different synaptic glutamate receptors across conditions of univesicular and multivesicular release, the transition in signaling that occurs for physiological conditions that span LTP and LTD, and the relative change in NMDAR-based synaptic signaling that occurs during synaptic maturation, when synapses shift from containing nearly all NR1/NR2B-NMDARs to a mix of either NR1/NR2A or NR2A/NR2B-NMDARs.

### Sensitivity to glutamate diffusion rate

**Figure 2:**
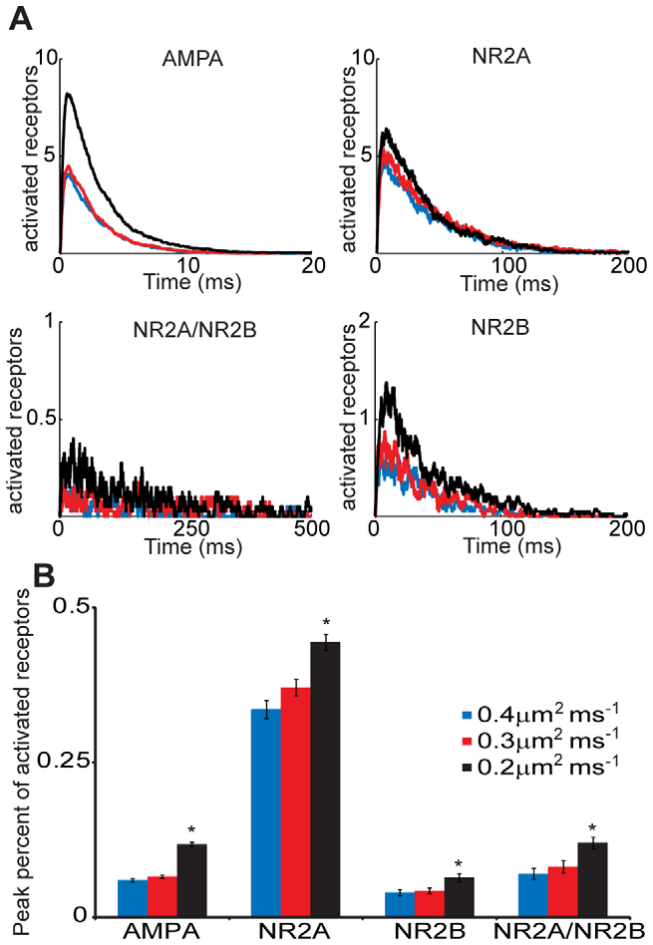
Differential sensitivity to diffusion rate among glutamate receptors. (A) Activation of synaptically localized 80 AMPARs and 20 NMDARs of single subtype was observed in response to a release of 3000 glutamate molecules with differing glutamate diffusion rates (0.2 µm^2^ ms^−1^, black; 0.3 µm^2^ ms^−1^, red; 0.4 µm^2^ ms^−1^, blue). (B)The peak percent of open receptors was predictably decreased for all receptors at higher glutamate diffusion rates. AMPARs displayed the most sensitivity of diffusion rate between 0.2 and 0.4 µm^2^ ms^−1^, while NMDAR subtypes all behaved similarly. (* p<0.05 compared to 0.4 µm^2^ ms^−1^).

We first sought to examine the sensitivity of receptor activation to the glutamate diffusion rate (D_glu_). Published estimates on the effective glutamate diffusion rate have varied from 0.2 to 0.76 µm^2^ ms^−1^ [34] and it is likely that this variation can affect the extent of activation among AMPARs and NMDAR subtypes. Similar to previous models [28], [41] we populated the postsynaptic face of the spine with 80 AMPARs and 20 NMDARs of a single type - NR1/NR2A-NMDARs, NR1/NR2B-NMDARs, or triheteromeric NR1/NR2A/NR2B-NMDARs. Activation was observed after a point release of 3000 glutamate molecules with varied D_glu_, 0.2–0.4 µm^2^ ms^−1^, a range of commonly used rates in recent models [28], [34], [42], [43]. Predictably, the general trend for all receptors was increased numbers of activated receptors for the slower diffusion rates (Figure 2A). Quantified, the peak percent of activated receptors after glutamate release was significantly greater at D_glu_ = 0.2 µm^2^ ms^−1^, for AMPARs and all NMDAR subtypes (p<0.05 compared to 0.4 µm^2^ ms^−1^) (Figure 2B). Interestingly, AMPAR activation was the most sensitive to D_glu_, producing the largest percent change among receptors, while all NMDAR subtypes had similar sensitivities. This suggests that while D_glu_ may effectively scale NMDAR activation, the relative patterns of activation among subtypes is unaffected. Thus, with the understanding that D_glu_ can impact receptor activation, all subsequent simulations were conducted with a rate of 0.2 µm^2^ ms^−1^, a rate which is reported to account for molecular obstacles and overcrowding [43]. To provide a direct comparison between the subsequent simulations and earlier studies of AMPAR and NMDAR activation [28], [35], [36], [40], we used the same kinetic parameters for the receptor activation scheme as used in these previous studies.

### Dynamic range of activation for synaptic glutamate receptors

**Figure 3:**
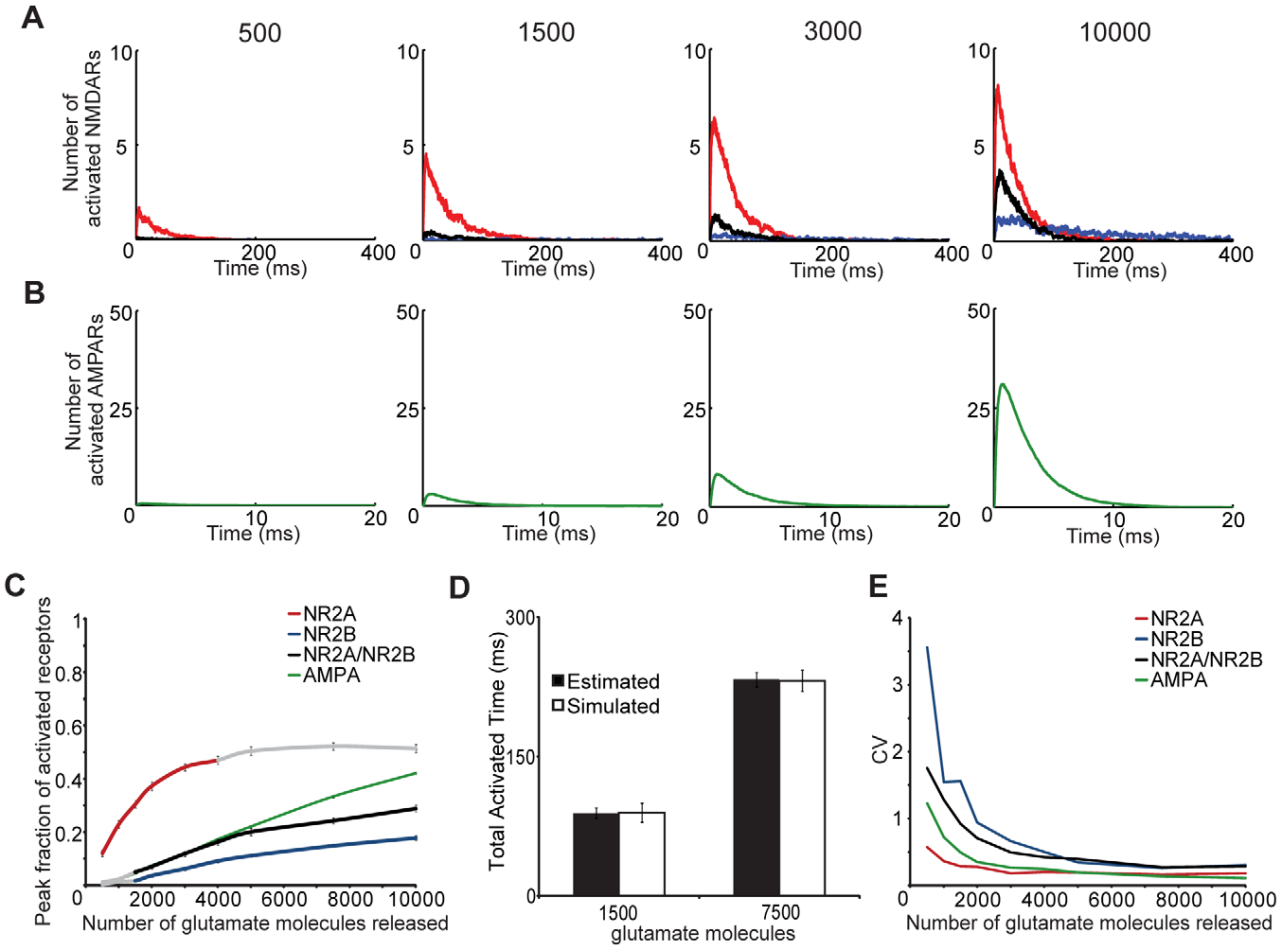
Dynamic range of NMDAR subtype and the scalability to a physiological synapse. Activation or opening of each receptor subtype was observed in response to varied levels of glutamate release (500–10,000 molecules, number denoted above graphs in A). Number of activated (A) NMDA receptors over time is shown for NR2A (red), NR2B (blue), and NR2A/NR2B (black) and (B) AMPA receptors in response to 500, 1,500, 3,000, and 10,000 molecules. (C) NR2A-NMDARs show a significantly higher fraction activation compared to the other subtypes. NR2A-NMDAR activation increases (p<0.05) over the physiological range of univesicular glutamate release (500–1,500 molecules), but saturates at larger glutamate levels (shaded in gray), while activation of NR2B-containing NMDARs significantly increases only in the range of multivesicular glutamate release (2,000–10,000) (p<0.05). Colored segments represent regimes of increased activation compared to preceding release amount (p<0.05). Peak percent of AMPARs significantly increases over the entire range of modeled glutamate release (p<0.05). (D) Average response of an individual receptor for each subtype can be used to estimate the total synaptic response of a mixed population of receptor subtypes (8 NR2A, 8 NR2A/NR2B, and 4 NR2B) that is not different from responses observed when the activation of this mixed population is indeed simulated. (n = 40 simulations per condition) (E) The stochastic variation in response, reflected in the coefficient of variance calculated for receptor opening, is greatest for NR2B-containing NMDARs at low levels of glutamate release, but variation is decreased for each glutamate receptor subtype at large numbers of released glutamate.

Our next objective was to define how either a single or near simultaneous release of multiple glutamate vesicles from the presynaptic bouton would activate AMPARs and NMDARs on the postsynaptic surface. Again, the postsynaptic face of the spine with 80 AMPARs and 20 NMDARs of a single type - NR1/NR2A-NMDARs, NR1/NR2B-NMDARs, or triheteromeric NR1/NR2A/NR2B-NMDARs. Physiologically, the size and glutamate concentration of synaptic glutamate vesicles can vary, with approximate glutamate content of 500–1,500 molecules [44], [45]. Across this entire range of glutamate release conditions, the concentration of glutamate in the synaptic cleft decayed rapidly to less than 10% of its peak value within 3–5 milliseconds. AMPAR peak activation significantly increased throughout the entire range of released glutamate (Figure 3C), ranging from 0.8%+/−0.1% (mean +/− standard error) at 500 molecules to 42.1%+/−0.4% at 10,000 molecules. The AMPA response showed no noticeable saturation across the range of glutamate release conditions tested, indicating this receptor population will show a dynamic scaling across the entire range of simulated conditions.

In contrast to AMPAR response, the activation of different NMDAR subtypes was influenced strongly by the amount of initial glutamate release. The peak percent of activated receptors increased most rapidly with NR1/NR2A-NMDARs, in agreement with results from Santucci and Raghavachari [36]. Across conditions modeling univesicular release, a scaled NMDAR response occurred only with NR1/NR2A-NMDARs. After a release of 500 molecules, mean peak percent of activated receptors was 12.1%+/−1.1% for NR1/NR2A-NMDARs, 1.3%+/−0.4% for triheteromeric NMDARs, and 0.4%+/−0.2% for NR1/NR2B-NMDARs, compared to 30.6%+/−1.4%, 5.1%+/−0.8%, and 1.8% +/−0.5%, respectively, after release of 1,500 molecules (Figure 3C). In conditions approximating multi-vesicular release, NR1/NR2A-NMDAR activation saturates, with no additional significant increase after 4,000 molecules. In comparison, activation significantly increases for both NR1/NR2B-NMDARs and triheteromeric NMDARs; from 1,500 to 10,000 molecules, the activation of NR1/NR2B-NMDARs steadily increases from 1.9%+/−0.5% to 17.9%+/−0.9%, while the NR1/NR2A/NR2B-NMDARs increase from 5.1%+/−0.8% to 29%+/−1.3%. Together, these simulation results show three behavior regimes for NMDARs – an initial phase dominated by NR1/NR2A-NMDAR activation, followed by a second phase that includes contribution of all NMDAR subtypes, and a third phase where scaling of the synaptic NMDAR response is prominently driven by the NR1/NR2B-NMDARs.

Although these results illustrate the behavior of subtypes in isolation, they do not provide a realistic picture of the synaptic composition that appears over time in cultured neurons, or in different brain regions. Rather than using a large number of simulations to examine all possible combinations of NR2A, NR2A/NR2B, and NR2B NMDARs at a physiological synapse, we tested if predictions from a proportional scaling of the response from individual receptor subtypes would match simulations of synapse populated with a mixture of different NMDAR subtypes. We computed the average activation time for an individual receptor for each subtype, scaled this proportionally for the number of these receptors appearing at a ‘mixed’ synapse, and produced estimates of the total synaptic activation time from a single, glutamate release event. Our ‘mixed’ synapse included 8 NR1/NR2A-NMDARs, 8 NR1/NR2A/NR2B-NMDARs, 4 and NR1/NR2B-NMDARs. Proportional scaling estimates of the total activation time for a release of 1500 and 7500 glutamate molecules were not statistically different from stochastic simulation of the same ‘mixed’ synaptic formulation (Figure 3D). These results indicate that it is possible to use the response of individual subtypes to correctly predict the synaptic response to a diverse set of receptors.

### Fidelity of the synaptic response is receptor dependent

These simulations also provide information on the consistency or fidelity of the synaptic response. We define the fidelity of the response as the variance in the numbers of receptors activated for a specified number of glutamate molecules released from the presynaptic bouton. As expected, an increase in the amount of glutamate released leads to a decrease in the calculated coefficient of variance (CV) for the postsynaptic AMPAR response. In general, the CV for all receptors asymptotically decreased at larger levels of released glutamate (Figure 3E), and the range of the predicted stabilized CV is within the range of similar measures reported for dissociated hippocampal neurons and slice cultures (0.2–0.6) [46]. Each receptor type showed a different transition point for achieving a stable synaptic signal response. For simulations releasing more than 5,000 glutamate molecules, there was no significant reduction in the CV for the AMPAR response. Similarly, the CV of the NR1/NR2A-NMDAR did not change significantly when more than 3,000 molecules were released. The NR1/NR2B-NMDARs produced the most variable response, with a relatively large CV calculated for the univesicular release conditions and stable CV achieved for simulations releasing more than 5,000 glutamate molecules. Together, these simulations show that NR1/NR2A-NMDARs provide the largest dynamic range and highest signaling fidelity under conditions of univesicular release, and AMPARs provide a somewhat smaller dynamic range and more variability across the same conditions. At higher levels of glutamate release, the AMPARs retain their dynamic range and improve their fidelity of signaling. Conversely, the NR1/NR2B-containing NMDARs show a more usable dynamic range and improvement in signaling fidelity under multivesicular release conditions.

### NMDAR subtypes show distinct temporal activation receptor ‘flickering’ behavior

**Figure 4:**
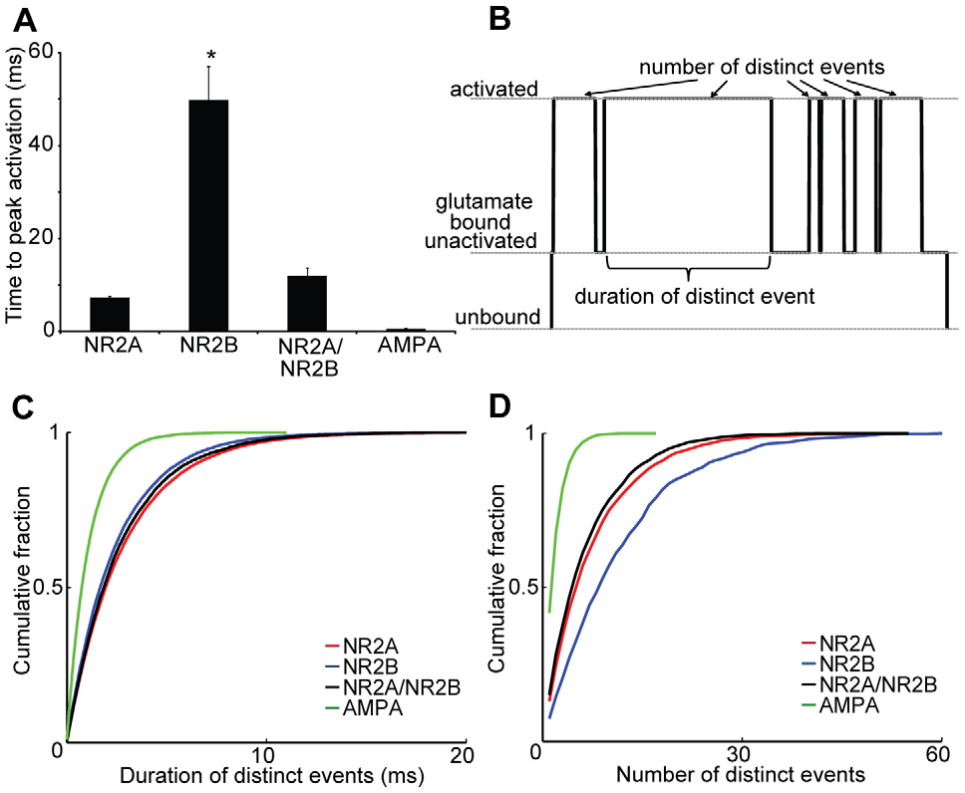
Slower kinetics and increased receptor flickering prolong NR2B activation. The activation events for all NMDARs were analyzed to discern differences in the temporal activation patterns among subtypes. (A) NR2B-NMDARs reach peak activation significantly slower than NR2A-NMDARs and NR2A/NR2B-NMDARs (* p<0.05 NR2B vs NR2A and NR2B vs NR2A/NR2B). (B) Receptor “flickering”, defined by the ability for a receptor to have multiple activation events without glutamate unbinding was analyzed using cumulative distributions to (C) demonstrate that NR2A-NMDARs (red) have significantly longer durations of individual events compared to NR2B-NMDARs (blue), triheteromeric NMDARs (black) and AMPARs (green) (KS test - p<0.01). (D) However, NR2B-NMDARs have significantly more distinct events per binding compared to other subtypes (KS test - p<0.01).

Both the magnitude and timing of glutamate receptor activation are key parameters that contribute to the type and extent of resultant signaling. The time to the peak activation of AMPARs following the initial release of glutamate was shortest among the studied glutamate receptors, indicating these receptors are well suited as rapid event detectors for glutamate release. Interestingly, the rise time was not significantly different between NR1/NR2A-NMDARs and NR1/NR2A/NR2B-NMDARs (mean +/− SE; 7.37+/−0.30 ms (NR1/NR2A) vs. 12.07+/−1.66 ms (NR1/NR2A/NR2B)). Based partly on the affinity of glutamate for the NR2B subunit, the time to peak activation of the NR1/NR2B-NMDARs is significantly slower than all other glutamate receptor types (49.9+/−7.2 ms; p< 0.01; Figure 4A). Once opened, the NR1/NR2A-NMDARs remained open longer than either the NR1/NR2A/NR2B or NR1/NR2B-NMDARs before transitioning to a bound, closed state (Figure 4B,C) (Kolmogorov–Smirnov test, p<0.01). All NMDARs showed a significantly longer initial activation period than AMPARs (p<0.01).

Following the initial activation and opening of each glutamate receptor subtype, all studied receptors showed a stochastic switching between the bound/open and bound/closed state or ‘flickering’ of the receptor (Figure 4D) before dissociation of glutamate from the receptor subunit. Simulations show that NR1/NR2B-NMDARs have more flickering events per glutamate binding than NR1/NR2A-NMDARs and NR1/NR2A/NR2B-NMDARs (Figure 4E,F). For the NR1/NR2B-NMDARs, these results explain why, despite the shorter receptor activated time, NR1/NR2B mediated calcium currents typically have a slower decay than NR1/NR2A mediated currents [16],[47]. Again, the diverse responses of these subtypes better allow for unique postsynaptic currents at synapses populated with a diverse set of receptors.

### A shift in the pattern of NMDAR subtype activation occurs with stimulation frequency

Activation of NMDARs is a major mediator in several models of synaptic plasticity, including LTP and LTD. Recently, conflicting evidence has emerged on the specific role of distinct NMDAR subtypes for certain types of plasticity [20], [21], [48], [49]. We used our simulations to evaluate glutamatergic signaling and observed the activation patterns of NMDAR subtypes in response to various frequencies of presynaptic stimulation. For these simulations, the spine model was populated with physiologically relevant numbers and localizations of NMDAR receptor subtypes: 8 synaptic NR1/NR2A-NMDARs, 8 synaptic triheteromeric NMDARs, 4 synaptic NR1/NR2B-NMDARs, and 10 extrasynaptic NR1/NR2B-NMDARs. Using common stimulation protocols in the literature [50] presynaptic stimulation was varied from 5 Hz–100 Hz and lasted for 1 second. Presynaptic glutamate release was stochastically determined using a recent model of presynaptic vesicular release dynamics [51]. All simulations were performed with a uniform synaptic vesicle content (1,500 glutamate molecules).

**Figure 5:**
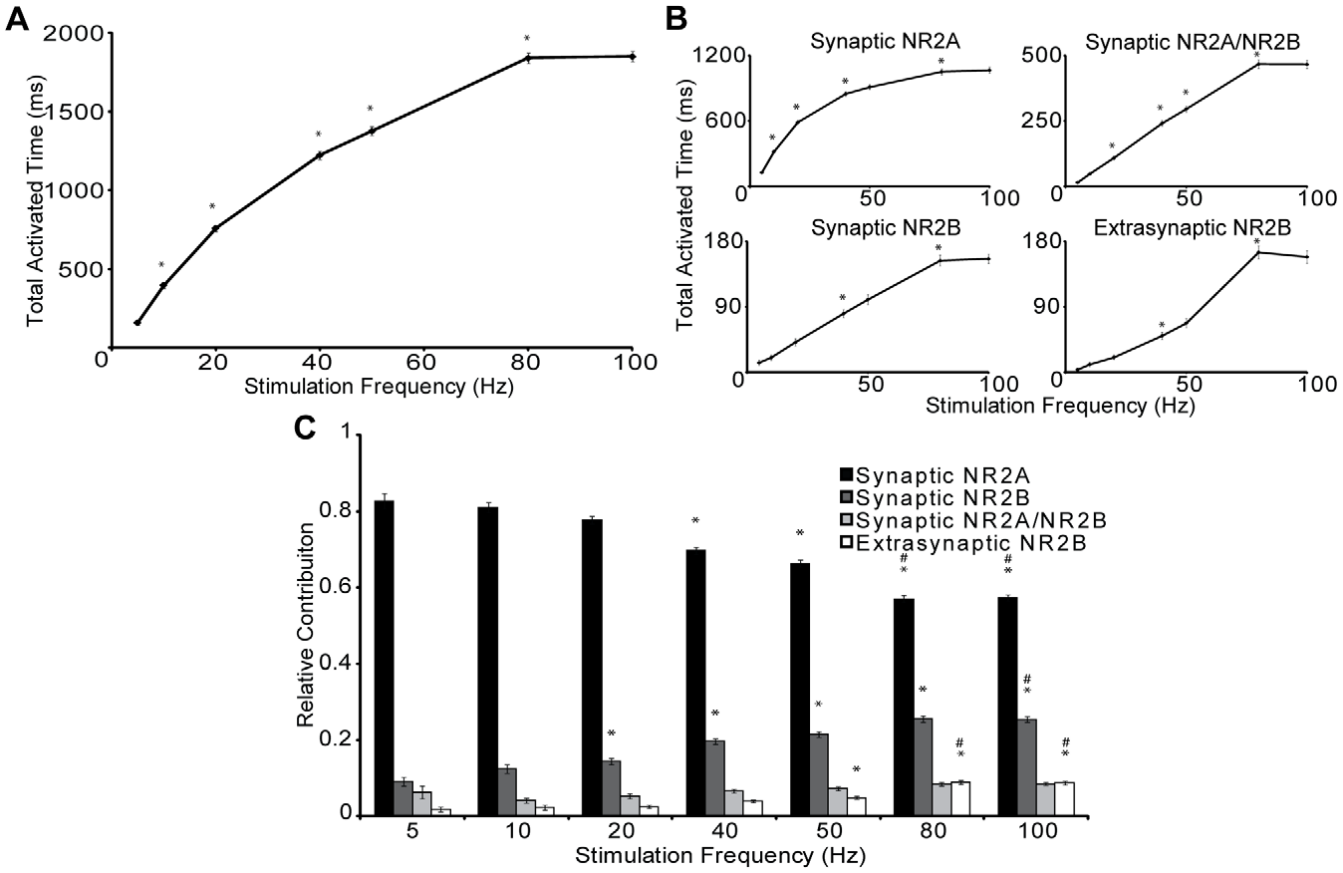
Frequency mediated shifts in NMDAR subtype activation patterns. The dendritic spine model (80 AMPARs, 8 NR1/NR2A NMDARs, 4 NR1/NR2B NMDARs, 4 NR1/NR2A/NR2B, and 10 extrasynaptic NR1/NR2B NMDARs) was subjected to presynaptic stimulation of various frequencies (5 Hz–100 Hz), and the stochastic release of glutamate vesicles was simulated using an approach developed for hippocampal synapses [Kandaswamy et al, 2010]. Total activated time increases for (A) all NMDARs and (B) for each NMDAR subtype individually as the stimulation frequency is increased (* p<0.05 significant increase from previous frequency). (C) Relative contribution for each subtype to the total receptor open time shows the changing patterns of NMDAR subtype activation during frequency stimulation, with NR2A-NMDAR contributing less and NR2B containing NMDARs contributing more at high frequency stimulations. (* p<0.05 compared to contribution at 5 Hz, # p<0.05 compared to 50 Hz) (n = 100 simulations per condition).

The period of NMDAR activation increased significantly across most of the stimulation frequency range, showing saturation above 80 Hz. Activation of the NR1/NR2A-NMDARs at the synapse increased most rapidly at low stimulation frequencies (<25 Hz), tapering slightly beyond 5 Hz. In comparison, synaptic NR1/NR2B-NMDARs, synaptic NR1/NR2A/NR2B-NMDARs, and extrasynaptic NR1/NR2B-NMDARs showed a linear increase in activation over nearly the entire stimulation range. Although the total activation time is dominated by NR1/NR2A-NMDARs at all frequencies, its contribution significantly decreased while the contribution of other subtypes, including NR1/NR2A/NR2B-NMDARs and extrasynaptic NR1/NR2B-NMDARs, significantly increased at higher stimulation frequencies (Figure 5C). This finding demonstrates that the activation patterns of NMDARs differ across stimulation frequencies, suggesting potential NMDAR subtype dependent mechanisms for different modes of synaptic plasticity. As NMDAR subtypes are known to activate different signaling pathways, increasing contribution of NR2B containing NMDARs at higher frequency stimulation may alter the balance of subtype specific signaling, inducing long term synaptic changes.

**Figure 6:**
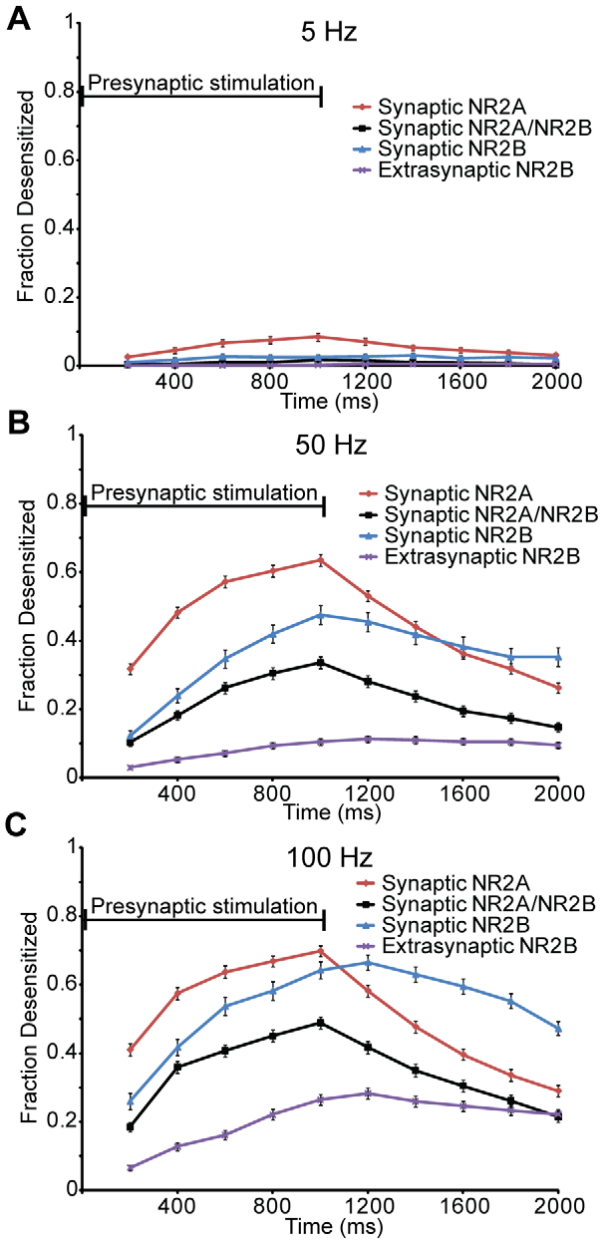
NR2A-NMDARs desensitize and recover faster than other subtypes. The fraction of receptors that are desensitized over a one second stimulation, and one second post stimulation, were recorded for each NMDAR subtype. (A) At 5 Hz, only a small portion of NMDARs is desensitized, these are primarily NR2A-NMDARs. At (B) 50 Hz and (C) 100 Hz, NR2A-NMDARs have a significantly greater fraction of desensitized receptors compared to all other subtypes, while synaptic NR2B-NMDARs show a significantly greater fraction of desensitized receptors compared to triheteromeric NMDARs (p<0.05).

To investigate why NR1/NR2A-NMDAR contribution decreases at higher frequencies, we examined the extent of receptor desensitization for each receptor subtype both during and after the presynaptic stimulation. Following a 5 Hz presynaptic stimulation, only a small fraction of receptors desensitized, and most of these desensitized receptors are NR1/NR2A-NMDARs (Figure 6A). After a 50 Hz and 100 Hz stimulation (Figure 6B,C), a significantly larger fraction of NR1/NR2A-NMDARs become desensitized compared to other subtypes, primarily due to their higher probability of glutamate binding. Moreover, synaptic NR1/NR2B-NMDARs exhibit a significantly larger fraction of desensitized receptors compared to triheteromeric NR1/NR2A/NR2B-NMDARs and extrasynaptic NR1/NR2B-NMDARs at higher stimulation frequencies, indicating the triheteromeric and extrasynaptic NMDARs may play an important role in sensing a sustained, bursting behavior in networks. In contrast, the NR1/NR2A-NMDARs and NR1/NR2A/NR2B-NMDARs recover faster from receptor desensitization compared to other subtypes (recovery at 1 sec post 100 Hz stimulation- NR1/NR2A-NMDARs: 58.5%, NR1/NR2A/NR2B-NMDARs: 56.0%, synaptic NR1/NR2B-NMDARs: 26.5%, extrasynaptic NMDARs: 15.9%), suggesting these receptor subpopulations may provide a mechanism to detect repeated interval bursts in a network.

### Developmental changes in synaptic NMDAR content alters synaptic calcium influx

Given the diversity of the postsynaptic response over both stimulation frequency and receptor composition, we sought to explore the potential differences in NMDAR synaptic signaling that can occur over development, as well as in disease states. The content of NMDARs at synaptic sites is highly dependent on neuronal age, with a developmental switch from predominantly NR2B expression early to increased NR2A expression later in development [16], [17], [18]. Moreover, brain injury may cause a change in the balance of NMDAR composition [52], yet the effect of this change on synaptic signaling is largely unknown. To this end, we used the flexibility of these computational simulations to explore the potential diversity in synaptic signaling that can occur during synaptic maturation.

Several experimental models of LTP appear in the literature. In the previous section (Figure 5), we simulated the most well established protocol for LTP induction (100 Hz, 1 sec duration) [53]. In comparison, other common models include chemically-induced LTP and spike timing dependent plasticity (STDP). To extend our findings and develop testable predictions for *in vitro* studies, we used our computational model to examine the role of subtype content in simulated chemically-induced LTP.

To examine how the identity of synaptic NMDAR subtypes can influence overall receptor activation, we simulated several possible configurations of synaptic NMDAR content. We compared activation across spines populated with different mixtures of NMDAR subtypes - from synaptic NMDARs consisting of only NR1/NR2B-NMDARs, to simulate a spine in early development before NR2A expression, up to and including synaptic NMDAR content of only NR1/NR2A-NMDARs as a representation of the canonical ‘mature spine’. In all cases, the number of synaptic NMDARs was kept at 20, and the number and identity of AMPARs and extrasynaptic NR1/NR2B-NMDARs were constant. Finally, we computed the net calcium influx that occurred during each simulation, using techniques to account for the magnesium block of the receptor and AMPAR-induced depolarization of the spine (see Methods for more details).

**Figure 7:**
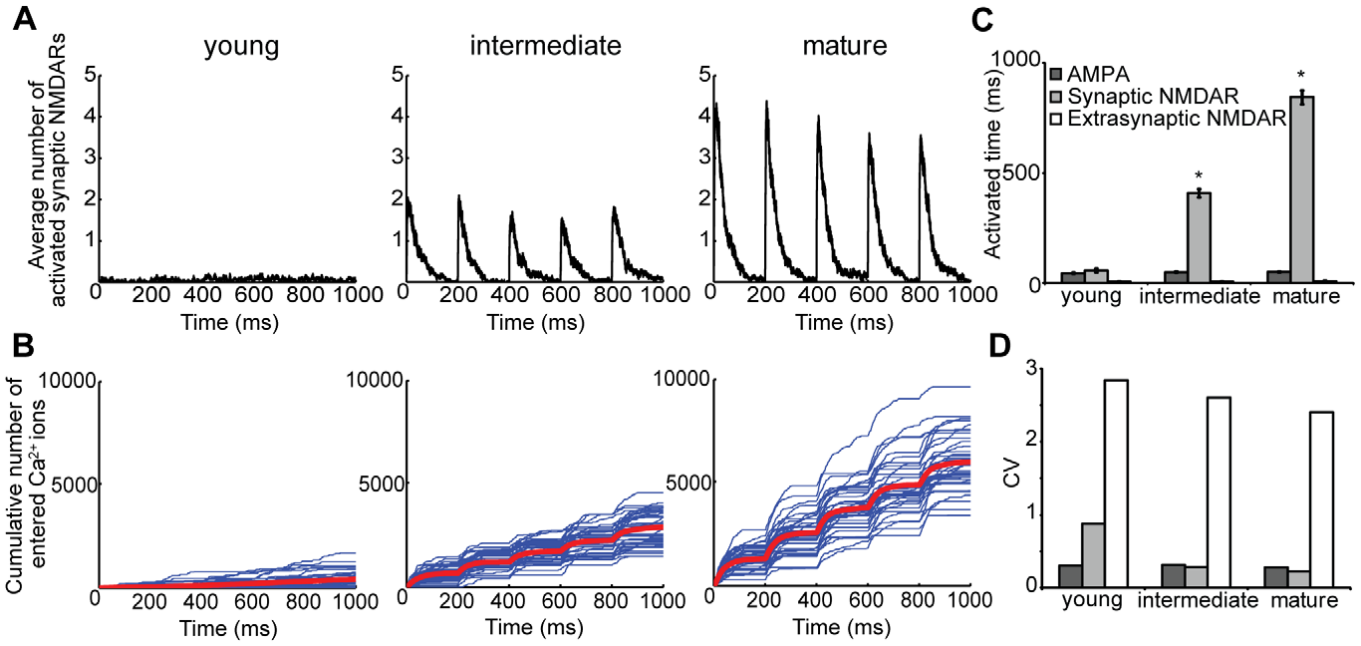
Increasing synaptic NR2A content during development enhances response and improves fidelity. Induction of chemically induced late phase LTP was simulated with a 5 Hz frequency of glutamate release on three different representations of synaptic NMDAR content; ‘young’ (20 NR2B-NMDARs), ‘intermediate’ (8 NR2A-NMDARs, 8-NR2A/NR2B-NMDARs, 4 NR2B-NMDARs), and ‘mature’ (20 NR2A-NMDARs). (A) Traces of the average number of activated synaptic NMDARs over all simulations and (B) cumulative calcium entry (blue: individual simulations, red: averaged over all simulations) demonstrate that younger cultures, dominated by NR2B, result in less predicted calcium influx. (C) Quantification of total activated time and (D) its coefficient of variance show that changes in relative synaptic NMDAR subtype content occurring through development result in significantly greater activation and less variance, suggesting that NR2A content is a major driving force in the reliability and magnitude of downstream signaling (* p<0.05 compared to other distributions, n = 40 simulations per condition).

Chemically induced LTP relies on a sustained period of action potential bursts that propagate through the network, where the duration of each burst can last for 1–3 seconds and the frequency of measured synaptic responses within each burst is approximately 5 Hz [54], [55]. Our simulation results show that 5 Hz glutamate release results in significant differences that occur in the NMDAR-mediated signaling in the spine across these different NMDAR subtype configurations. As expected, observed synaptic NMDAR activation was significantly reduced in a model representation of the immature spine (i.e., 100% NR1/NR2B-NMDARs) when compared to spines with a physiological mix of NMDAR subtypes and with more mature representations (Figure 7A,C). Increasing the fraction of NR1/NR2A and NR1/NR2A/NR2B NMDARs increased significantly the ability to elicit a defined synaptic NMDAR activation during simulated chemical LTP induction. Furthermore, predicted calcium influx through the NMDAR was significantly different in all three configurations suggesting that synaptic content can significantly impact the resultant signaling from this stimulation (Figure 7B). Additionally, we found the reliability of synaptic NMDAR activation is increased through maturation, as measured by the observed decrease in coefficient of variance of synaptic NMDAR activation (Figure 7D). Our data suggests that the NR2A content of the synapse is the major driving force in both the reliability and extent of the NMDAR response and provides a potential mechanism to age dependent functional outcomes.

**Figure 8:**
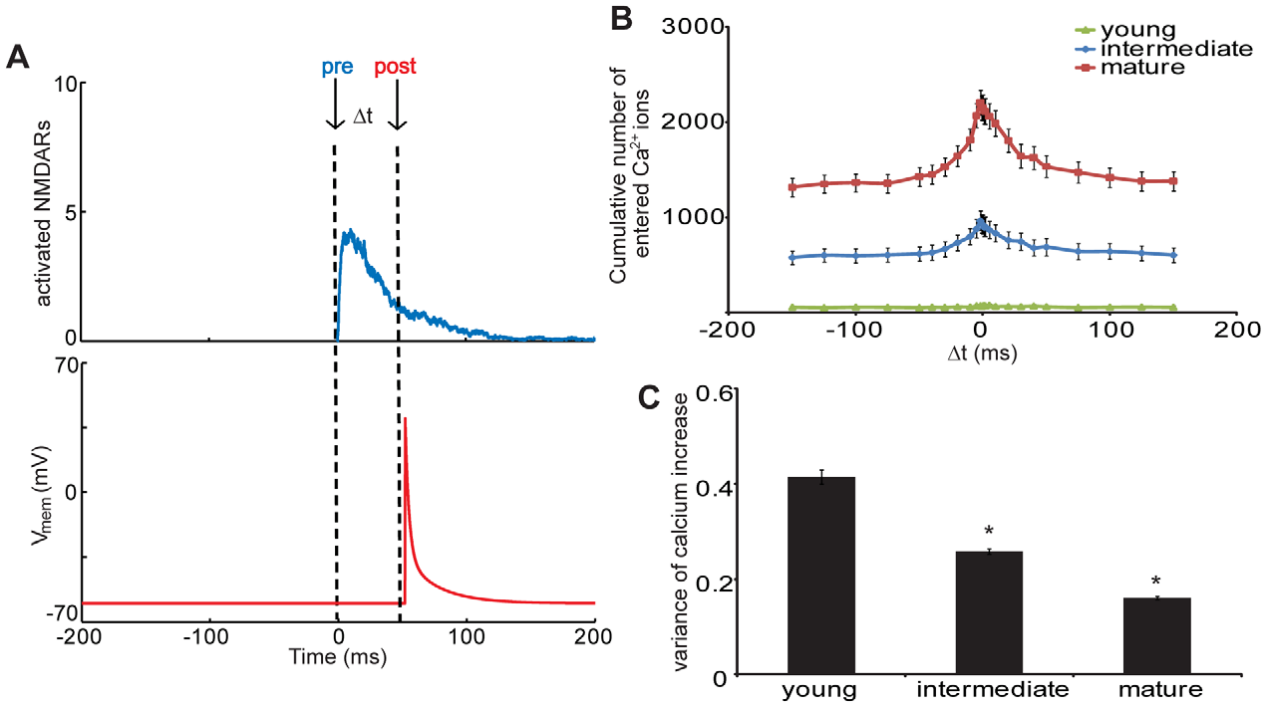
Changing subtype content during development improves calcium potentiation during paired stimulations. (A) Spike time dependent plasticity (STDP) was simulated by pairing presynaptic glutamate release with postsynaptic depolarization at different time intervals. (B) Calcium influx was greatest in the mature subtype content and was increased by paired stimulation when pre and post spikes were given simultaneously (Δt = 0). (C) The variance in the fold increase of calcium influx generated by paired depolarization was greatest for young cultures, again demonstrating that NR2A content significantly improves the extent and reliability of signaling during this model of plasticity (* p<0.05 compared to other distributions).

STDP relies on the precise timing of presynaptic and postsynaptic stimulation, with the time interval between stimulation defining the potential for long term synaptic changes [56], [57]. Thus, we computed calcium influx at the different developmental NMDAR subtype content configurations at distinct time intervals (Δt) between presynaptic glutamate release and postsynaptic depolarization. Depolarization, modeled as an immediate increase in membrane potential with a slow hyperpolarizing tail [58], induces a transient relief of the Mg^2+^ block which, dependent on receptor activity during depolarization, can potentiate calcium influx caused by the presynaptic spike (Figure 8A). We demonstrate that postsynaptic spikes significantly potentiates influx in our model of the intermediate and mature spine, with greatest increase at Δt = 0, whereas influx was not significantly potentiated at young configurations. As demonstrated previously, activation of synaptic receptors is significantly decreased at NR2B dominated young configurations, and thus calcium influx increases as NR2A content increases (Figure 8B). Interestingly, the maximal fold increase of calcium entry, compared to conditions without a postsynaptic spike, was similar for both intermediate and mature spine configurations at approximately 1.7, suggesting that the ability for spike timing to potentiate initial calcium influx holds for varying subtype content. However, the variance in the fold increase is significantly smaller in mature conditions (Figure 8C), again demonstrating that NR2A content improves the fidelity of NMDAR signaling.

**Figure 9:**
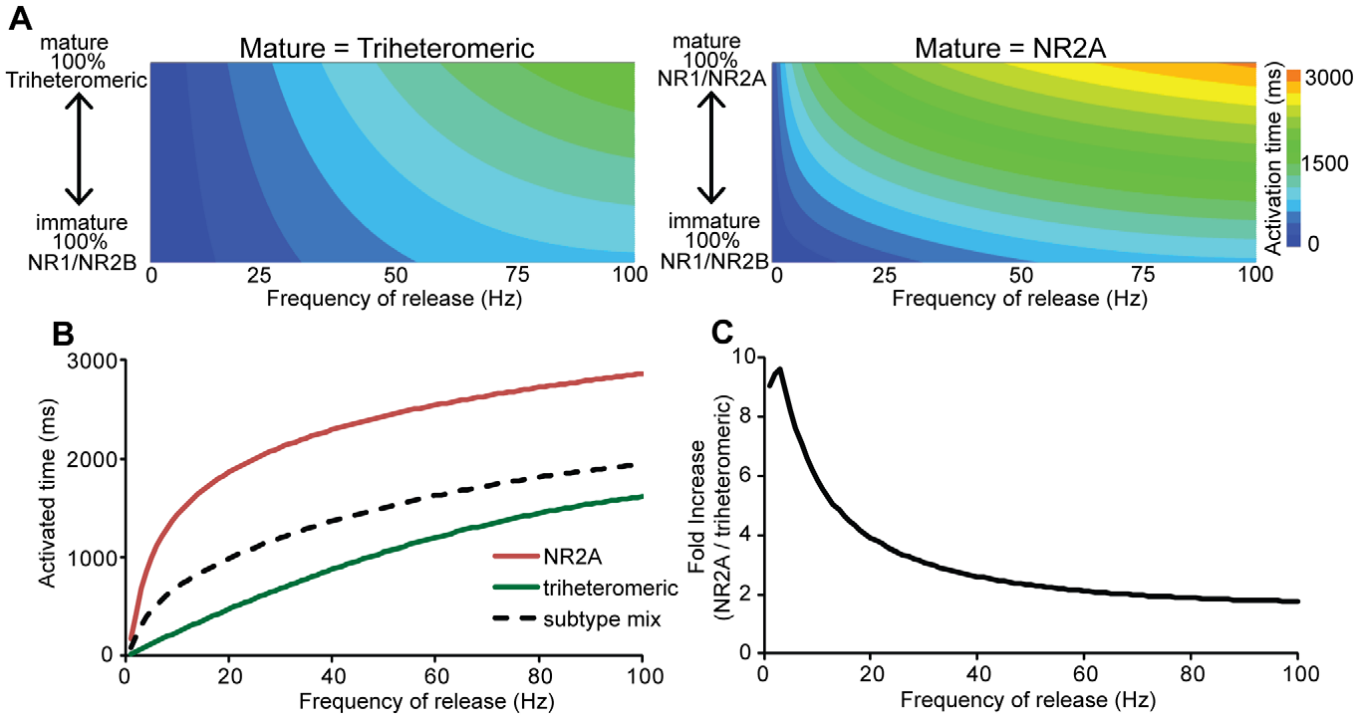
Presence of NR1/NR2A-NMDAR in the ‘mature’ subtype preferentially enhances calcium influx at low frequency stimulation. Synaptic responses were calculated from changing configurations of synaptic content simulating spine maturation, where the ratio of NMDARs was alternatively adjusted from (immature) all NR2B to (mature) either all NR2A or NR2A/NR2B. (A) Distribution of responses demonstrates that transitioning to a triheteromeric ‘mature’ state provides more stability in synaptic response through maturation process, particularly at lower stimulation frequencies. (B) Activation of the mature states compared to synaptic mix of subtypes, as defined by reported PSD content [100] demonstrate how subtype content influence overall activation. The enhancement in activation time was especially evident at low stimulation frequencies (C), where the relative increase in NR2A-NMDAR synapses was almost ten times the response of synapses containing only triheteromeric NMDARs.

Finally, we estimated the synaptic NMDAR response to glutamate release at different frequencies for two alternative views of NMDAR content at a mature spine. Some have suggested that in the mature brain, NMDARs are dominated by the triheteromeric NR1/NR2A/NR2B-NMDAR subtype [59], [60]. Others suggest a mixture of NR1/NR2A and triheteromeric NMDARs [61]. To study the possible range of responses, we used average frequency dependent responses per receptor to calculate the total synaptic NMDAR activated time at spines in which the ratio of synaptic NMDARs was alternatively adjusted between NR1/NR2B-NMDARs and NR1/NR2A-NMDARs or, alternatively, between NR1/NR2B-NMDARs and triheteromeric NMDARs. We found that the developmental transition to a triheteromeric mature state provides more stability in the postsynaptic responses through the spine maturation process (Figure 9A). Moreover, the most dramatic difference between the two views of the mature spine appears at low frequency stimulation, where the activation at the NR2A mature spine was approximately 10 times greater compared to the triheteromeric mature spine (Figure 9C). This suggests that calcium-sensitive processes are likely particularly sensitive to the identity of the ‘mature’ subtype in low frequency conditions, and NR1/NR2A-NMDAR content at a mature synapse enhances the ability to distinguish between different types of low frequency glutamate signals. Interestingly, the relative difference between the two views of the mature spine is less significant at higher stimulation frequencies (Figure 9B) where the proportional change in activation between higher frequency stimulation is not different between the two synaptic representations.

## Discussion

In this report, we utilize a computational model of glutamatergic signaling at a single excitatory synapse to study activation patterns of specific NMDAR subtypes during spontaneous and coordinated neurotransmission. The importance of NMDAR subtype in neuronal signaling is widely recognized, with differences in kinetics, localization, and developmental regulation among NMDAR subtypes shaping the influence and timing of signals to promote survival, programmed cell death, and even the local activation of signaling networks within individual spines [62], [63], [64]. Our current work builds upon previous computational models of glutamatergic signaling by investigating the role that NMDAR subunit composition plays in synaptic transmission across a broad physiological range including univesicular, multivesicular, and repeated glutamate release events that occur when a burst of action potentials arrive at the presynaptic bouton. Three new aspects emerge from our current work. First, each NMDAR subtype shows a distinct dynamic range before saturation, highlighting how the varied composition of the individual NMDAR subtypes at single spines can significantly shape the postsynaptic response. Second, the relative contribution of each NMDAR subtype changes across different input stimulation frequencies, with an increased diversity of receptor activation occurring at higher stimulation frequencies. Finally, the developmental expression of NMDARs impacts signaling through NMDARs across all physiological conditions, with immature synapses showing relatively modest activation compared to more mature synapses. Coupled with the knowledge that NMDAR composition can vary over development, these simulations suggest that a single physiological process, such as either LTD or LTP, may have distinct regulating mechanisms that change throughout development and partly explain the existing confusion surrounding the role of NMDAR subtypes on single neuron, as well as neuronal network, function.

### Subunit-specific dynamic range of NMDAR activation

Our data illustrates that NR1/NR2A-NMDARs are robustly activated when single vesicles within the physiological range of glutamate content (500–1,500 molecules) [44], [45] are released. For the geometry we studied, the NR1/NR2A-NMDARs represent the only significant and reliable component of the NMDAR population activated across this physiological range of glutamate vesicles. Past reports suggest that variability among individual vesicles can represent an important source of variation in postsynaptic responses of AMPARs [29], [65], [66]. Our work shows that NR1/NR2A-NMDARs share a similar ability to vary the postsynaptic response, also in direct proportion to the number of glutamate molecules in the vesicle. Moreover, this variation in response occurs with relatively high fidelity; the coefficient of variance for NR1/NR2A-NMDAR activation across the univesicular range is approximately 4–6 times less than either the NR1/NR2A/NR2B or NR1/NR2B receptors. Therefore, among the NMDARs at the synapse, the NR1/NR2A-NMDARs appear ideally suited to detect a vesicular release event, and to scale this detector response in proportion to the amount of glutamate released from the vesicle. The consistency of NR1/NR2A-NMDAR activation under spontaneous release conditions – i.e., its ability to detect discrete, synaptic release events - may facilitate the pro-survival role of synaptic NMDARs, the preferential location for NR1/NR2A-NMDARs [63], [67]. Indeed, a smaller number of studies highlight the specific and important role of NR1/NR2A-NMDARs in mediating pro-survival signaling [68], [69], in pathological conditions. Therefore, maintaining the activation of synaptic NMDARs across a broad range of conditions appears to be an ideal advantage of NR2A-containing NMDARs. The unique advantage of NR1/NR2A-NMDARs to ‘detect’ and ‘scale’ their response during univesicular release (UVR) is less clear. Graded responses in NMDAR activation will naturally produce proportional graded responses in secondary messengers including calcium, calcium bound calmodulin, and enzymes such as calpain, a protease directly activated by calcium binding. However, many intracellular signaling networks, including MAP kinase activation [70] and CaMKII phosphorylation [71], [72], function to convert graded signals into strong switch like signals. Thus, the graded response of NMDAR activation can produce similarly graded outcomes in some signaling pathways while also being used by other pathways to simply approach a critical threshold.

A notable shift in the dynamic range of NMDAR populations occurs with multivesicular release (MVR) conditions; the relative activation of NR1/NR2A-NMDARs saturates and the proportional activation property shifts to NR1/NR2B-NMDARs and NR1/NR2A/NR2B-NMDARs receptors. Similar to NR1/NR2A-NMDARs functioning as detectors during UVR conditions, this shift in the NMDAR activation pattern suggests NR2B-containing NMDARs are the primary detectors of MVR. It is important to note that MVR occurs at some, but not all types of synapses, with notable absence of MVR at mossy fiber – granule cell [73] and CA3 – interneuron connections [74], [75]. Furthermore, there has been great controversy on the presence of MVR at Schaffer collateral – CA1 synapses [65], [76], [77]. This variability indicates that, in the absence of other compensatory mechanisms, the role of NR2B in physiological signaling may be somewhat limited in MVR lacking synapses. Interestingly, our observed shift in the scaling of the NMDAR populations also occurs simultaneously with an improvement in the consistency or fidelity of signaling mediated through NR2B-containing NMDARs, as indicated by the lowered coefficient of variance (CV) predicted from the MVR simulations. This improvement in signaling fidelity may seem inconsistent with published reports, as multivesicular release is often reported with high values of CV calculated from miniature excitatory postsynaptic currents (mEPSCs) [78], [79], [80]. Our simulations indicate that in response to large, nonvariable numbers of glutamate molecules, the stochastic nature of NMDAR activation contributes little to the variability observed at high CV synapses. The high CV observed experimentally during MVR is instead likely mediated by presynaptic mechanisms, including vesicular glutamate content [81], [82] and number of vesicles released [30], [78], [79].

Perhaps most interesting is the transition or shift in the activation of different NMDAR populations at the synapse for MVR (also reported in Santucci and Ragavachari, 2008) that can significantly impact the type and extent of downstream signaling. More emphasis is placed in recent studies to discriminate among NMDARs, as specific NMDAR subtypes are tied to different and often opposing pathways [14], [22], [24]. For a synapse dominated by NR1/NR2B-NMDARs, our simulations suggest that MVR, or other compensatory mechanisms, is necessary to improve the consistency of the signaling mediated through the synaptic NMDARs. It is interesting to note that several studies cite the increased frequency of MVR in immature neuronal cultures, where the expression of NR1/NR2B-NMDARs dominates [79], [83]. Alternatively, if a synapse contains a majority of triheteromeric NMDARs, the synapse would have a broadened ability to respond more consistently to both UVR and MVR, although this synapse would still have limited ability to reliably detect NMDAR signaling for small, single vesicles containing less than approximately 1,000 molecules. In this synaptic configuration, commonly described for mature synapses, the synapse would show the broadest operating range for NMDAR signaling. Moreover, the insertion of NR1/NR2A-NMDARs into a synapse clearly provides ability to detect even more subtle single vesicle release events, and offers a dramatic improvement in the fidelity of signaling compared to either the NR1/NR2B or NR1/NR2A/NR2B-NMDARs (approximately 4∶1) over the range of single vesicles containing 500–1,500 glutamate molecules. To this end, past work shows that NMDAR trafficking will show a preference for inserting NR2A-containing NMDARs when the NMDAR activity is suppressed for a significant period of time, or when the selective activity of synaptic NMDARs is suppressed [84], [85].

An equally important consideration for NMDAR-mediated signaling is the gradual activation of extrasynaptic NR1/NR2B-NMDARs, an event that is unlikely for the release of single vesicles or low frequency stimulation, but is more probable for MVR and high frequency stimulation. A number of studies now show the relative balance between synaptic and extrasynaptic NMDARs is important for determining the net resultant role for NMDARs, e.g., the sustained activation of NR2B-containing NMDARs are linked to activation of p38 MAPK [86], inhibition of pro-survival transcription, and swelling of neuronal mitochondria [12], all of which and contribute to neuronal death. Among the receptor populations analyzed, the extrasynaptic NR1/NR2B-NMDAR exhibits the lowest probability of activation, and their relatively sparse number indicates they will not significantly contribute to the predicted overall NMDAR current. This does not exclude the possibility that these receptors can contribute meaningfully to the response across the physiological range, as only the brief activation of extrasynaptic NMDARs has been reported to alter PKC activation and AMPAR subunit composition [87] as well as having a role in LTD induction [21]. However, the kinetics and localization of extrasynaptic NMDARs make it well suited for the transduction of excitotoxic signals in pathological conditions. Together, these simulations suggest a tight regulation of synaptic transmission is necessary to ensure the proper health of the neuronal network. In addition, the multiple subtypes of NMDARs and their differential dynamic ranges allows for a single mature synapse to be able to receive and transmit various types of physiological glutamate signals into appropriate intracellular signaling pathways.

### The role of NMDAR subtypes in synaptic plasticity are influenced by synaptic content

Experimentally, Liu et al. and others [20], [21], [88] show that low frequency stimulation (5 Hz) mediates a long-term synaptic depression dependent on NR2B-containing NMDARs, and not on the activation of NR1/NR2A-NMDARs. However, others report that NR2B is not essential for LTD [89]. Our simulations show that neither synaptic nor extrasynaptic NR1/NR2B-NMDARs contribute significantly to the total NMDAR activation observed under low frequency stimulation, seemingly in agreement with NR2B playing no role in LTD. However, as the low frequency stimulation for LTD is applied over several minutes (typical duration 10–15 minutes) [50], one clear possibility is that the modest and sustained activation of the NR1/NR2B-NMDAR over several minutes will integrate to activate the signaling necessary to trigger LTD. An alternative possibility is if elements of the LTD signaling pathway were localized to the macromolecule signaling domains of the NR1/NR2B-NMDAR, where even low levels of NMDAR activation would produce sufficient calcium influx to activate molecules within a highly localized signaling complex near individual NMDARs. In this condition, the local activation of the NR1/NR2B-NMDAR would be relatively insensitive to the more robust activation of the NR1/NR2A-NMDAR. Nanodomain-mediated signaling for NMDARs is receiving more attention lately, as this local activation is capable of changing synaptic AMPAR number [22], composition, and the relative activation of MAPK signaling modules in the spine [22], [90]. One intriguing possibility is the direct physical interaction of NR2B with Ras-GRF1 and SynGAP, required for the successful activation of p38 MAPK [90] and inhibition of ERK [22], respectively. Both pathways result in reduced AMPAR surface expression and LTD induction - therefore raising the possibility that LTD may be partly influenced by nanodomain-signaling mediated by NR1/NR2B-NMDAR activation.

The role of the NMDAR subtype on the induction of LTP is widely debated, with several reports suggesting that it is dependent on NR2A [20], [21], on NR2B [19], [48], or that both subunits are involved [23], [49], [91]. Our simulations show that there is a distinct shift in the patterns of NMDAR subtype activation for higher frequency stimulations; the contribution of NR1/NR2A-NMDARs is significantly decreased, while the contribution of synaptic NR1/NR2A/NR2B-NMDARs and extrasynaptic NR1/NR2B-NMDARs is significantly increased. Certainly, one straightforward explanation for the NMDAR-dependent threshold of LTP is that higher frequency stimulation simply activates more NDMARs, and this more significant activation of the NMDARs will lead to a shift in the intracellular signaling that favors LTP. This argument suggests that the induction of LTP is dependent on overall global increase in calcium, a commonly cited mechanism for regulating LTP [92], [93]. An alternative explanation, though, is that the LTP is triggered by a transition in the activation of more NR2B-containing NMDARs for higher frequency stimulations [48], [94], a prediction borne out in our simulations. There is support for both possibilities in the literature. Several reports demonstrate that LTP induction is mediated by an overall calcium load [49], [95], [96], while others have identified specific NMDAR subtype specific signaling complexes that can control LTP [90], [97]. Furthermore, a recent report shows that both NR2A and NR2B containing NMDARs can induce LTP, but use distinct signaling pathways [23]. Our simulations suggest that the composition of the NMDARs at the synapse is a key factor that can influence the relative likelihood for each proposed mechanism. For example, a synapse dominated by NR1/NR2B-NMDARs will produce relatively modest calcium influx and therefore increase the importance of physically localized signaling complexes. Alternatively, mechanisms relying more on global increases in calcium would apply more prominently in a maturing synapse containing a higher fraction of NR2A-containing NMDARs. A key experimental tool needed to test these possibilities is specific inhibition of each NMDAR pool, a tool that remains elusive [27]. Once such a tool is available, our simulation studies of different stimulation protocols and receptor content will provide guidance in investigating exactly how NMDAR subtypes and overall calcium load influence activation of intracellular signaling pathways and initiation of long term synaptic changes associated with synaptic plasticity.

Together, our data demonstrates the unique properties of NMDAR subtype specific activation, and shows how subtypes may be suited for specific roles in NMDAR signaling. Further, we illustrate the patterns of NMDAR activation can change under different glutamate release conditions, during different developmental states, and that receptor content is an important factor in the reliability of NMDAR signaling. The unique properties of these subtypes provides flexibility to synaptic transmission allowing efficient transfer of different types of glutamate signals into distinct patterns of NMDAR subtype activation. Future simulations in concert with experimental investigations will be vital in the understanding of regulatory mechanisms at the synapse and how they impact observed diversity in NMDAR function.

## Methods

### Geometry and receptor content

We modeled spine geometry as a typical thin spine with an octagonal-shaped spine head (500 nm diameter) and long spine neck. We represented the postsynaptic face as a 300 nm × 300 nm square, separated by 20 nm from an identically shaped presynaptic face [39]. A membrane surrounded the entire presynaptic bouton and postsynaptic spine head, also separated by a 20 nm distance from the apposing surfaces. Glutamate receptors randomly decorated the postsynaptic surface using previous estimates of NMDA and AMPA receptor density along the postsynaptic surface for CA1 neurons (80 AMPARs, 20 NMDARs) [98], [99]. To examine differences in activation parameters among NMDAR subtypes, simulations used a uniform composition of receptors along the postsynaptic face, represented with either 20 NR1/NR2A-NMDARs, 20 NR1/NR2B-NMDARs, or 20 NR1/NR2A/NR2B-NMDARs. Based on the relative amounts of NR2A and NR2B shown to be localized within the postsynaptic density (PSD) [100], we developed another distribution for some simulations, where the 20 synaptic NMDARs were divided into 8 NR1/NR2A-NMDARs, 8 NR1/NR2A/NR2B-NMDARs and 4 NR1/NR2B-NMDARs. As previous reports show that approximately 30% of all NMDARs are located extrasynaptically [101], we placed 10 extrasynaptic NR1/NR2B-NMDARs randomly along the sides of the spine head.

### Glutamate release

Glutamate was released in the synaptic cleft as a point source near the center of the face of the presynaptic bouton. Both univesicular and multivesciular release profiles were simulated. Single vesicles of glutamate ranged from 500–1,500 molecules, as defined by previous reports [44], [45]. We modeled multivesicular release using the simultaneous release of a larger number of glutamate molecules (2,500–10,000) in the cleft, assuming an available releasable pool of 5–20 vesicles in the hippocampal synapse [102]. A limited set of simulations showed that the release of a large number of glutamate molecules from the center of the cleft did not produce results significantly different from simulations using multiple release of individual vesicles (data not shown).

In simulations of varied frequency stimulus trains, presynaptic stimulation (5–100 Hz for 1 second) was modeled to generate random glutamate vesicle release profiles, defined by the calculation of frequency dependent release probabilities (Pr) [51]. Briefly, this model utilizes stimulus trains to calculate presynaptic facilitation and augmentation, two calcium dependent components which influence the probability of vesicle release. Additionally, the state and recovery of two glutamate vesicle pools, the readily releasable pool and recycling pool, are observed to account for vesicle rundown during the stimulus. Frequency dependent parameters (personal communication, V. Klyachko) were thus used to generate Pr at each individual spike which, along with the state of the readily releasable pool, was used to determine if each spike resulted in a released vesicle. Distinct vesicle release profiles were generated for 100 simulations per frequency, each of which was applied to our dendritic spine model with a physiologic representation of NMDAR subtypes.

### Glutamate receptor state modeling

Glutamate binding and activation of AMPARs and NMDAR subtypes was modeled by implementing previously published reaction schemes (Figure 1). The AMPAR activation model of Jonas et al. includes the binding of two glutamate molecules and three receptor desensitized states [40]. NMDAR activation was modeled using the reaction scheme of Erreger et al., which contains specific reaction rates for both NR2A-NMDARs and NR2B-NMDARs (Table 1) [35]. This scheme includes the binding of two glutamate molecules as well as a dual stage activation and two desensitized states which occur after glutamate binding. The reaction scheme for triheteromeric NMDARs was developed by modeling glutamate binding to both a NR2A and a NR2B subunit and using reaction rates that were averages of the rates for NR2A and NR2B (personal communication – K. Erreger) [36].

**Table 1:** Reaction rates used for AMPAR and NMDAR subtype activation.

		NR2A-NMDAR [35]	NR2B-NMDAR [35]	NR2A/NR2B-NMDAR [36]	AMPAR [40]
**k_on_**	(nm^3^ ms^−1^)	52,456	4,698		
**k_on-A_**	(nm^3^ ms^−1^)			52,456	
**k_on-B_**	(nm^3^ ms^−1^)			4,698	
**k_off_**	(ms^−1^)	1.010	0.0381		
**k_off-A_**	(ms^−1^)			1.010	
**k_off-B_**	(ms^−1^)			0.0381	
**k_s+_**	(ms^−1^)	0.230	0.048	0.139	
**k_s−_**	(ms^−1^)	0.178	0.230	0.204	
**k_f+_**	(ms^−1^)	3.140	2.836	2.988	
**k_f−_**	(ms^−1^)	0.174	0.175	0.1745	
**k_d1+_**	(ms^−1^)	0.0851	0.550	0.318	
**k_d1−_**	(ms^−1^)	0.0297	0.0814	0.0556	
**k_d2+_**	(ms^−1^)	0.230	0.112	0.171	
**k_d2−_**	(ms^−1^)	0.00101	0.00091	0.00096	
**k_R-RA_**	(nm^3^ ms^−1^)				7619.4
**k_RA-R_**	(ms^−1^)				4.260
**k_RA-RA2_**	(nm^3^ ms^−1^)				47,144
**k_RA2-RA_**	(ms^−1^)				3.260
**k_RA2-O_**	(ms^−1^)				4.240
**k_O-RA2_**	(ms^−1^)				0.900
**k_RA-D1_**	(ms^−1^)				2.890
**k_D1-RA_**	(ms^−1^)				0.0392
**k_RA2-D2_**	(ms^−1^)				0.172
**k_D2-RA2_**	(ms^−1^)				0.000727
**k_O-D3_**	(ms^−1^)				0.0177
**k_D3-O_**	(ms^−1^)				0.004
**k_D1-D2_**	(nm^3^ ms^−1^)				2,108.2
**k_D2-D1_**	(ms^−1^)				0.0457
**k_D2-D3_**	(ms^−1^)				0.0168
**k_D3-D2_**	(ms^−1^)				0.1904

### Model parameters

Table 1 summarizes the rate constants used to describe receptor kinetics for AMPARs [40] and NMDAR subtypes [35]. All other important model parameters are summarized in Table 2. Our models used a glutamate diffusion constant of 0.2 µm^2^/ms^−1^ [34], which is on the lower end of the range of estimated glutamate diffusion constants that have been reported in the literature. All surface boundaries of the spine, presynaptic membrane, and surrounding neuropil membrane reflected glutamate molecules.

**Table 2:** Parameters used in model.

D_glut_	0.2 µm^2^ s^−1^ (unless otherwise noted) [34]
Number of AMPARs	80 [41]
Total number of synaptic NMDARs	20 [98], [99], [100]
Number of extrasynaptic NMDARs	10 [101]
Synaptic cleft width	20 nm [39]
Glutamate molecules per vesicle	1,500 (unless otherwise noted) [44], [45]

### Analysis

Simulations were carried out using Smoldyn 1.84, a spatial stochastic simulator for biochemical reaction networks [37], [38]. Smoldyn models biomolecular reactions by using reaction rates to compute binding radii and diffusion rates to determine spatial position of potential reactants. All simulations had time steps of 0.01 ms, based on a numerical convergence study showing that the simulations results did not differ between time steps of either 0.01 ms or 0.001 ms. Unless otherwise noted, simulations were terminated when the solution reached 1 second. The state of all available receptors (glutamate bound, open, glutamate unbound, etc), the number of receptors in each state, the location of all receptors, and the position of released glutamate molecules was tracked for all simulations. Post-processing of model results was performed with user-generated scripts developed in MATLAB (Mathworks, Natick, MA). Statistical significance among multiple group comparisons was found using ANOVA and posthoc Tukey's analysis. Analyzing receptor opening distribution profiles was accomplished using two-sample Kolmogorov-Smirnov tests to determine significance between cumulative frequency distributions.

### Calcium entry

Calcium entry into the spine was computed by using an iterative process to calculate change in membrane voltage potential (*V_m_*) and the probability for open NMDARs to be blocked by magnesium (Mg^2+^). We used the relationship established by Jahr and Stevens [103] to calculate the probability of each receptor to be blocked by magnesium at each time step, defined as
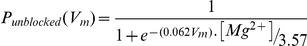
We assumed a magnesium concentration of 0.8 mM, and calculated *V_m_* at each time step by finding the incremental change in *V_m_* dictated by total ionic flux through AMPARs and NMDARs by
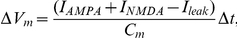
where *I_AMPA_*, *I_NMDA_*, and *I_leak_* are calculated using
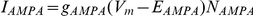


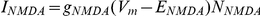


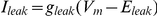

*N_AMPA_* and *N_NMDA_* are the number of open receptors of each receptor type. It was assumed that *g_AMPA_* and *g_NMDA_*, the single channel conductance for each receptor, was 12 pS and 45 pS respectively. The reversal potentials, *E_AMPA_* and *E_NMDA_*, for both AMPARs and NMDARs were assumed to be 0 mV. In computing a generalized leak current, a leak conductance, g_leak_, was assumed to be 10 nS, with a reversal potential of −60 mV. Finally, the membrane capacitance (*C_m_*) of the spine was found using a reported capacitance density of 1 µF/cm^2^ [104]. The probability for a receptor to be unblocked by magnesium (*P_unblocked_*) was then used to determine if each individual activated NMDAR, as defined by Smoldyn simulations, was able to conduct calcium in that time step. The number of calcium ions entered per open NMDAR per time step was calculated using a probability distribution of ions entered given by
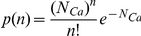
Here, *N_Ca_* is the average number of calcium ions entered and is computed by
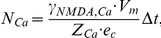
where the single channel calcium conductance for NMDARs, *γ_NMDA,Ca_*, is assumed to be 4.5 pS, *Z_Ca_* is the valence for Ca^2+^ (z = 2), and *e_c_* is the elementary charge (1.6×10^−19^C).
